# 

**Published:** 1830-10-01

**Authors:** 


### BIBLIOGRAPHICAL RECORD;

OR,

*Works received for Review, from the 25th June to the 25th September*, 1830.

1. Report of the Managing Committee of the House of Recovery and Fever Hospital, in Cork Street, Dublin, for one year, ending 4th January, 1830; with the Medical Report annexed. 8vo. pp. 112. Dublin, 1830.

---

2. A Treatise on the Pathology of the Animal Fluids and Solids. Consisting chiefly of a succinct account of the principles of Exclusive Solidism and Exclusive Humoralism, and an Inquiry into the Validity of each relatively, illustrated by cases. By W. STOKER, M.D. Dublin. 8vo. stitched, pp. 123, 1830.

---

3. A Treatise on the Mineral Waters of Harrogate, and its Vicinity. By ADAM HUNTER, M.D., &c. 8vo. pp. 138. 1830.

---

4. Traité de Chimie Appliquée aux Arts. Par M. DUMAS. Tome deuxieme. 8vo. pp. 808, 1830. Avec Planches.

---

5. A Series of Chemical and Medical Tables, forming a Synopsis of Chemistry, Materia Medica, Pharmacy, and Nosology. By JOHN HOGG, Surgeon, and Graduate in Medicine, &c. House-surgeon and Apothecary to the Dispensary of the University of London.

---

6. Rules and Regulations of the Metropolitan Society of General Practitioners in Medicine and Surgery, instituted May 1, 1830.

7. A concise Treatise on Dislocations and Fractures. Being a Selection from the most approved Foreign and English Surgical Authorities, from the days of Celsus to the present time. Illustrated by 14 Plates; 18mo. pp. 110. London, 1830. Price 4s. 6d.

*** *A very useful little compilation. We recommend it to our younger brethren desirous of acquiring information on this subject.*

---

8. An Introduction to the Practice of Midwifery. By the late Thomas Denman, M.D. Third American edition, with numerous Notes, Engravings, and Emendations. By J. W. FRANCIS, M.D. New York, 1829, pp. 776.

☞ *This is a very valuable re-publication of a Stock-book. We shall notice some of the notes in our Periscope.*

---

9. Appendix to a second edition of a Series of Observations on Strictures, &c. By R. A. STAFFORD. 8vo. pp. 52, 1830.

---

10. A Treatise on Pulmonary Consumption; its Prevention and Remedy. By JOHN MURRAY. Small 8vo. pp. 156. London, 1830.

---

11. Practical Remarks on the Nature and Effects of the expressed Oil of the Croton Tiglium, &c. By MICHAEL J. SHORT, M.D. 8vo. pp. 64, sewed. 1830.

---

12. Table of Vegetable Poisons, illustrated with Coloured Drawings. By G. SPRATT, Surgeon, Fellow of the Medico-Botanical Society of London, &c. stitched, two Tables and Plates. Wilson, London, 1830.

☞ *A very useful and ably executed table. We recommend it warmly.*

13. Remarks on the Disease called Hydrophobia; Prophylactic and Curative. By JOHN MURRAY, F.S.A. &c. &c. 8vo. pp. 86, Longman, 1830.

14. On Canine Madness; comprising the Post-mortem Appearances, Nature, Origin, and Preventive and Curative Treatment of Rabies in the Dog, and other Domestic Animals; being a Series of Papers published in the "VETERINARIAN," in 1828-9-30. By W. YOUATT, V.S. and F.Z.S., &c. &c. 8vo. sewed, pp.

15. An Account of the Varieties in the Arterial System of the Human Body. By P. H. GREEN, A.B. M.B. Trinity College, Dublin. Octavo, pp. 39; illustrated by Plates, 4s.

☞ *Every Anatomist—every Surgeon should possess this little volume. Varieties in the distribution of Arteries are said to exist once in every eight times. If so —these are somewhat more than exceptions to general rules.*

16. An Outline of the Sciences of Heat and Electricity. By THOMAS THOMSON, M.D. Regius Professor of Chemistry in the University of Glasgow, &c. &c. 8vo. pp. 583, with Wood-cuts. 1830.

17. An Introduction to Systematical and Physiological Botany. Illustrated with Explanatory Engravings. By THOMAS CASTLE, F.L.S., M.R.C.S., &c. 12mo. pp. 285, nine Plates. Cox, Borough, 1829.

☞ *This is a very valuable little work to the Students of Pharmacy and Medicine. To them we recommend it.*

18. Dissertatio Medica Inauguralis de Cholera. Auctore SAMUEL IRVINE, Hiberno, &c. &c. Edinburghi, 1830.

☞ *A very well written and instructive Thesis.*

19. A Rationale of the Laws of Cerebral Vision; comprising the Laws of single and erect vision, deduced upon the principles of Dioptrics. By JOHN FEARN, Esq. 8vo. pp. 176, London, 1830.

☞ *An erudite and original Essay, Metaphysical and Physiological.*

20. Disputatio Medica Inauguralis de Cholera Epidemica Indiæ Orientalis. Auctore GEORGIO WITT. Lugduni, Batavorum, 1830.

21. An Elemental Inquiry into the Structure and Functions of the Spleen. By WILLIAM DOBSON. 8vo. pp. 32. Wilson, 1830.

22. A Treatise on the Minute Anatomy of the Bones. By ANTONIO SCARPA. Translated from the last Latin edition. Duodecimo, pp. 70. Price 2s. 1830.

23. Practical Observations on Leucorrhœa, Fluor Albus, or "Weakness:" with Cases Illustrative of a New Mode of Treatment. By GEORGE JEWEL, Member of the Royal College of Surgeons, &c. 8vo. pp. 108. Wilson, 1830.

24. Traité Pratique sur les Maladies des Yeux; ou Leçons données á l'Infirmerie Ophthalmique de Londres, en 1825 et 1826, &c. Par W. LAWRENCE. Traduit par le Docteur C. BILLARD, &c. 8vo. Paris, 1830.

25. Two Memoirs read before the Royal Academy of Sciences at Paris, on the Successful Inhalation of Diluted Chlorine in the Early Stages of Pulmonary Consumption, &c. with Cases, &c. From the French of M. GANNALL. By W. H. POTTER, M.R.I., Operative Chemist. 8vo. pp. 93, 1830.

26. A Treatise on the Venereal Diseases of the Eye. By WILLIAM LAWRENCE, F.R.S. 8vo. pp. 337. Wilson, London, 1830.

27. A Short Tract on the Formation of Tumours, and the Peculiarities that are met with in the Structure of those that have become Cancerous; with their mode of Treatment. By Sir EVERARD HOME, Bart. 8vo. pp. 98. Longman, 1830.

28. On the Recent Improvements in the Art of Distinguishing the Various Diseases of the Heart, being the Lumleyan Lectures delivered before the Royal College of Physicians, in the year 1829. By JOHN ELLIOTSON, M.D. Cantab. &c. Folio, pp. 35, in double columns, with eight beautifully executed Plates. London, September, 1830.

### INDEX.

A.

Abercrombie, Dr. on stricture of the colon ........................ 150
Abercrombie, Dr. on the stomach and intestines .................... 223
Abortion, criminal, review on....... 97
Abortion, has it been spontaneous or not? ..................... 103
Abortion, English and French laws respecting ..................... 108
Abscess, sloughy, of the leg, report on 281
Abscesses opened by cupping-glass.. 565
Accumulations in the bowels, Mr. Annesley on................... 214
Acetate of morphia, Dr. Bardsley on 333
Acids and alkalies, influence of, on the blood ....................... 218
Acute rheumatism, quinine in..... 437
Adams, Dr. on tetanus............. 476
Addison, Dr. on female disorders .. 39
Adventitious structures, Dr. Hodgkin on......................... 44
Alcock, Mr. his lectures on surgery 24
Amaurosis, Dr. Knox on ........ 206
Amaurosis from debility, case of .. 209
Amaurosis from intemperance, case of 209
Amaurosis, treatment of.......... 211
Amaurosis treated by strychnine.... 441
Amaurosis, Mr. Middlemore on.... 460
Amaurosis, strychnine in.......... 460
Anatomical preparations in wax.... 192
Anatomical catalogue, Dr. Hodgkin's 367
Anatomy, difficulties of, in America, 499
Aneurism, cases of............... 276
Aneurism, popliteal, cases of...... 283
Aneurism, the "dissecting"........ 355
Aneurism, true & false, description of 357
Aneurism, surgical treatment of... 365
Aneurism, varicose, M. Dupuytren on 509
Aneurism by anastomosis......... 565
Aneurismal tumour, deceptive .... 569
Annesley, Mr. on the diseases of India 213
Anticyra, the mad-house of the Greeks 303
Aorta, aneurism of the .......... 179
Aorta, aneurisms of the .......... 187
Aorta, aneurism of the............. 277
Aortic aneurisms, treatment of.... 280
Aphonia and idiotcy from fright.... 512
Apoplexy of spleen, Mr. Ancill's case 247
Apothecaries' Company .......... 574
Arachnitis, with acute gastritis.... 158
Arachnitis, supposed cases of...... 169
Arteries, Mr. Guthrie on the ...... 133
Arteries, wounded, reparative processes in......................... 134
Arteries, modes of operating on.... 137
Arteries, wounded, M. Dupuytren on 139
Arteries, peculiar affection of the.. 180
Arteries, the deposites in, considered 279
Arteries, Mr. Guthrie on diseases of 350
Arteries, contractility of.......... 351
Arteries, erysipelatous inflammation of........................... 352
Arteries, changes of, in age........ 354
Arteries, preternatural, dilatation of 356
Arteries, collateral circulation of... 363
Auscultation, Dr. Elliotson on...... 425

B.

Bahama Islands, climate of the.... 78
Baillie, Dr. anecdote of .......... 489
Bardsley, Dr. his hospital facts.... 333
Barry, Dr. on the Gibraltar fever, 337
Beale, Mr. on deformities ........ 55
Bermudas, climate of the ........ 78
Blake, Dr. on delirium tremens.... 196
Bleedings, immense, in concussion, 561
Blind, eyes for them! ............ 481
Blood, Dr. Hall on the loss of ... 60
Blood, treatment of the loss of .... 67
Blood, Dr. Steevens on the......... 217
Blood, on the particles of the...... 371
Bloodletting in inflammation ...... 325
Bloodletting in irritation ........ 327
Bloodletting in fever ............ 412
Bowels, morbid matters in......... 214
Bowels, obscure affection of the.... 225
Brain fever, Dr. Burton Pearson on 198
Brain, opposite causes of disease in 316
Brain, mollescence of the ........ 500
Brain, anomalous symptoms referred to ......................... 560
British physicians, lives of......... 478
Bronchocele in Lower Canada .... 186
Bronchocele, influence of water, as a cause ........................ 373
Browne, Sir Thomas, on cremation 480
Buchanan, Mr. his ligature of the subclavian ...................... 501
Bunyon, Mr. Lawrence on...... .. 439
Burne, Dr. on stricture of the colon 228
Burrows, Dr., his letter .......... 147

C.

Calomel in cynanche tonsillaris.... 484
Cancer of the rectum, M. Lisfranc on 148
Cancer of the jaw—M. Delpech on.. 546
Canada, bronchocele in........... 156
Carbonate of iron, preparation of the 571
Carbuncle—gangrene of the stomach 545

Carditis, chronic, case of.......... 176
Carlisle, Sir Anthony, eyes for him 481
Carlisle, Sir Anthony, on the spleen 486
Carotid tied for hæmorrhage .... 263
Carotid tied for the same.......... 265
Carotids tied for aneurism........ 565
Cashin, Miss, inquest on.......... 572
Castration for diseased testis, case.. 457
Castration—French practice inferior to our's .................... 556
Catalepsy, Dr. Duncan's case of .. 250
Catalogue, anatomical, of Guy's Hospital ........................ 367
Cataract, report on .............. 239
Cataract, fluid, case of .......... 238
Cataract, soft, case of ............ 239
Cataract, congenital, cases of...... 239
Cataract, anomalous, case of...... 240
Cataract, capsular and hard, cases of 240
Caustic for hydrophobia.......... 436
Cava inferior, obliteration of the .. 550
Celibacy in those disposed to insanity 90
Cervix uteri, inflamed, symptoms of 420
Chaplains and veterinary surgeons, 113
Chest, report on the diseases of the 446
Children, M. Guersent on diseases of 552
Chlorine, as a preventive of hydrophobia ........................ 433
Cholera, Mr. Searle on............ 375
Choroid coat, Mr. Mackenzie on .. 253
Choroiditis, symptoms of ........ 253
Choroiditis, constitutional symptoms 255
Choroiditis, causes of ............ 256
Chronic arachnitis, supposed case of 169
Chronic inflammation of arteries... 353
Chronic disease of the testis ...... 393
Clark, Mr. on the carb. ferri ...... 571
Climate, Dr. Clark on............ 77
Climate of the Neilgherry ........ 308
Clouds, Dr. Thompson on ........ 475
Coagulation of the blood, in aneurismal sacs...................... 361
Coimbatoor;—climate of the ...... 308
Cold lotions to the chest.......... 153
Cold affusion, M. Recamier on .... 462
Collateral circulation of arteries.... 363
College of Surgeons.............. 232
Colon, stricture of sigmoid flexure of 150
Colon, cases of stricture of the..... 228
Coma from loss of blood .......... 62
Concussion of the brain, case of.... 233
Concussion, secondary symptoms, 234
Concussion, scalp wound.......... 235
Concussion, remarks on.......... 558
Concussion, treatment of ........ 561
Conolly, Dr. on insanity .......... 289
Contraction of the chest, case of .. 515
Convulsion from loss of blood .... 60
Convulsive epidemic....... ...... 571
Cooper, Sir Astley, on the testis.... 385
Corns, Mr. Lawrence on.......... 438
Coroner for Middlesex ............ 573
Coroner's inquest on St. John Long 521
Corrigan, Dr. on the heart ...... 122
Costello, Mr. on lithotrity ........ 257
Counter irritation in diseases of the heart ........................ 535
Cranial temperament ............ 14
Cremation, Sir Thomas Browne on.. 480
Criminal abortion, review on ...... 97
Cruveilhier, M. on fungous tumours 494
Cupping-glass for emptying abscess 565
Curative effects of loss of blood.... 323
Cynanche tonsillaris.............. 260
Cysts, simple serous, characters of.. 45

D.

Danger of suppressing habitual evacuations ........................ 467
Dangers of operations for deformities 505
Debility a cause of hysteria........ 31
Debility, remarks on ............ 314
Delirium from loss of blood ...... 61
Delirium tremens, Dr. Blake on .. 196
Delirium tremens, Dr. B. Pearson on 198
Delpech, M. on cancer of the jaw .. 546
Delpech, M. on imperforate os uteri 553
Denman, Dr. his letter to Dr. Parry 479
Depositions in the coats of arteries 352
Diabetes, Dr. Venables on........ 68
Diabetes, morbid anatomy of...... 71
Diabetes, treatment of .......... 73
Diaphragm, neuralgia of the...... 461
Difficulties of anatomy in America.. 499
Dilatation of arteries ............ 356
Dinners, medical, ancient and modern 489
Diploma of London University .... 204
Discovery of the use of the spleen.. 486
Diseases of arteries, Mr. Guthrie on 133
Diseases of arteries, Mr. Guthrie on 350
Diseases of the chest, report on.... 446
Diseases of the lungs report on .... 513
Diseases of the heart............ 535
Dislocated head of humerus ...... 464
Dislocation, congenital, of radius .. 464
Dissecting aneurism, description of 355
Distention of intestines .......... 151
Dobson, Mr. on the spleen ........ 531
Double bladder, supposed case of .. 160
Dublin Fever Hospital, report from 408
Duncan, Dr. his case of catalepsy.. 250
Dupuytren, M. on wounded arteries 139
Dupuytren, M. criticised by Mr. Guthrie ........................ 142
Dupuytren, M. on union by first intention ...................... 190
Dupuytren, M. on varicose aneurism 508
Dura mater, fungus of the...... .. 496
Dysmenorrhœa.................. 336

E.

Earle, Mr. his case of hæmorrhage 266

East India Company, memorial to the 114
Eccentricity, curious examples of .. 292
Ectropion, new operation for...... 566
Effusion, serous.................. 171
Elliotson, Dr. on the heart........ 424
Empyema, cases of ............... 447
Empyema, paracentesis thoracis for 449
Empyema, remarks on............ 452
Enema, opiate, poisoning by ...... 563
Erethismus ebriositatis, Dr. Blake on 196
Erysipelatous inflammation . .... 352
Evacuations, suppressed with danger 467
Excision of joints, Mr. Syme on the, 273
Excision of a carious rib, fatal .... 554
Exhaustion, with sinking ........ 64
Exostosis cured by mercury ...... 456
Expectoration of pus, mysterious .. 567
Extraction of a foreign body in larynx 465
Eyes for the blind, Mr. Adams'.... 481

F.

Falx, fungous tumour of the ...... 496
Fawdington, Mr. on nævus........ 506
Female disorders, Dr. Addison on.. 39
Femoral artery, ligature of the.... 276
Femoral artery tied, cases of...... 283
Fever, sources of, at Gibraltar .... 52
Fever, Dr. Smith's letter on ...... 230
Fever, Dr. Hall on loss of blood in 324
Fever at Gibraltar, contagiousness of, considered .... .......... 343
Fever and cholera, resemblances 379
Fever hospital, Dr. O'Brien's report 408
Fever, typhoid, types of .......... 409
Fever, state of the brain in........ 411
Fœtus, modes of proving it to have lived ........................ 104
Foreign body in trachea .......... 464
Foreign body in larynx, extracted.. 465
Fraser, Mr. his critique on Dr. Barry 337
French machines for the spine .... 58
Fright, idiotcy from............... 512
Functional disorders, Dr. Philip on 311
Fungous tumours of meninges .... 494
Fungus hæmatodes of the lung .... 189
Fungus hæmatodes of the liver.... 226
Fungus hæmatodes of the lungs.... 514

G.

Gangrene of the left upper extremity 180
Gastritis, acute, with arachnitis .. 158
General practitioners, dinner of the 192
General practitioners, society of the 270
George the Fourth, illness and death of ......................... 468
George the Fourth, post-mortem examination of .................. 470
Gibraltar, medical topography of .. 49
Gibraltar fever, critique of Dr. Barry on ......................... 337
Gibraltar fever, treatment of...... 348
Glaucoma, Mr. Mackenzie on...... 491
Gonorrhœa, Mr. Travers on ...... 162
Gonorrhœa, nitrate of silver in .... 424
Gooch, Dr. his dream ............ 478
Gooch, Dr. notice of him.......... 490
Guersent, M. on the diseases of children ......................... 552
Guthrie, Mr. on the arteries ...... 133
Guthrie, Mr. on the diseases of arteries ......................... 350
Guy's Hospital, anatominal catalogue of ....................... 367

H.

Hæmorrhage, modes of stopping .. 134
Hæmorrhage from the throat...... 144
Hæmorrhage, fatal, from the spleen 227
Hæmorrhage from the throat...... 260
Hæmorrhage, syncope from ...... 463
Hæmorrhage, secondary.......... 501
Halford, Sir Henry, Dr. Burrows' letter to ...................... 147
Hall, Dr. on the loss of blood...... 60
Hall, Dr. on loss of blood ........ 323
Harvey, memoranda respecting.... 498
Head, injuries of, report on ...... 233
Head, on injuries of the .......... 557
Heart, Dr. Corrigan on the ...... 122
Heart, diseases of the ............ 176
Heart, *simple* diseases of the ...... 317
Heart, Dr. Elliotson on the....... 424
Heart, M. Larrey on the ......... 535
Heart, marvellous disease of the .. 538
Hemeralopia, or night blindness .. 214
Hennen, Dr. on the Mediterranean 49
Hennen, Dr. on the state of Gibraltar 339
Hepatic abscess—curious case .... 567
Hernia humoralis, symptoms of.... 389
Hodgkin, Dr. on adventitious structures ...................... 44
Hodgkin, Dr. his catalogue ...... 367
Homer, Dr. his case of gastritis.... 158
Hooping-cough, Mr. Alcock on.... 29
Hook, the inventor of auscultation, 426
Hospital facts and observations .... 333
Hough, Mr. on the Neilgherry .... 308
Human horns .................. 567
Human mind, differences of the .. 10
Hunter, Dr. life of ............. 483
Hunterian operation inapplicable to wounded arteries .............. 138
Hydatid disease of testis.......... 400
Hydrophobia, Murray & Youatt on, 433
Hydrothorax, curious case of...... 569
Hypertrophy of the stomach ...... 491
Hypochondriasis, Dr. Reid on .... 83
Hysteria, Mr. Tate on............ 30
Hysteria, heroic treatment of...... 564

I.

Idiosyncrasy and temperament.... 2

Idiotcy from fright .............. 512
Imagination, portrait of the ...... 291
Imperforate vagina—fatal operation 466
Imperforate os uteri—operation for, 552
Impulse of the heart ............ 124
Indian diseases, Mr. Annesley on .. 213
Inferior cava, obliteration of...... 550
Inflammation simulated by hysteria 32
Inflammation of the pericardium, 183
Inflammation of the choroid ...... 253
Inflammation, on the loss of blood in 325
Inflammation, simulated by irritation 331
Inflammation, acute, of testis...... 388
Injuries of arteries, Mr. Guthrie on, 133
Injuries of the head, report on .... 233
Injuries of the head .............. 557
Inquest on St. John Long ........ 520
Insanity, Dr. Conolly on.......... 289
Intemperance, powerful remarks on 87
Intermittents, treatment of ...... 154
Internal strangulations .......... 544
Internal strangulations ............ 568
Iodine in leucorrhœa ............ 423
Irish epidemics, want not the cause 408
Iritis, Mr. Middlemore on ........ 459
Irritable testis .................. 397
Irritation of the uterus.......... 40
Irritation, loss of blood in ........ 327

J. & K.

Jaw, M. Delpech on cancer of the.. 546
Jenner, Dr. notices of............ 489
Jewel, Mr. on leucorrhœa ........ 416
Johnson, Dr. his case of phthisis .. 529
Joints, Mr. Syme's excision of..... 273
Kennedy, Dr. on life and mind.... 145
King George the Fourth .......... 468
Knox, Dr. on amaurosis ......... 206

L.

Larrey, M. on the heart .......... 535
Larrey, M. on the testis .......... 540
Laryngeal phthisis, case of........ 543
Laryngitis, cases of .............. 173
Larynx, foreign body in, extracted.. 465
Lawrence, Mr. on corns .......... 438
Leadenhall-street, medical tyranny 121
Leg, report on sloughy abscess of the 281
Leucorrhœa, &c. Mr. Jewel on .... 416
Leucorrhœa, actual seat of........ 417
Life and mind, Dr. Kennedy on.... 145
Ligature for popliteal aneurism.... 286
Lisfranc, M. on cancer of the rectum 148
Lithotrity, Mr. Costello on........ 257
Liver, on morbid affections of the.. 320
Lives of British physicians........ 478
Lives of British physicians........ 488
Lizars, Mr. on the division of nerves 271
London University, diploma of .... 204
Loss of blood, Dr. Hall on ........ 323
Louis, M. on pericarditis.......... 429
Lower jaw, case of cancer of ...... 550
Lower jaw, removal of great part of, 555
Lucid intervals in insanity........ 91
Luke, Mr. his case of hæmorrhage 263
Lunar influence ................ 485
Lunatics, Dr. Conolly on ........ 306
Lung, scirrhus of the ............ 520
Lungs, Dr. Philip on the diseases of, 318
Lungs, on malignant diseases of the, 513

M.

Machinery, spinal, varieties of .... 57
Macilwain, Mr. on stricture ...... 193
Mackenzie, Mr. on choroiditis .... 253
Mackenzie, Mr. on glaucoma .... 491
Madame Montmorenci .......... 57
Mad-house treatment of madness .. 302
Madness, detection and treatment of, 301
Madness, the dungeon and jacket for, 304
Malignant tumours.............. 47
Malignant diseases of the lungs.... 513
Man-midwifery, Sir Anthony's horror of .................. 482
Mayo, Mr. on ulceration of throat.. 265
Mechanical apparatus, Mr. Beale's, 55
Medical topography of the Mediterranean .................... 49
Medical department of East India Company .................. 112
Medical ranks, repletion of the.... 112
Medical diploma of London University ...................... 204
Medical Transactions of Paris .... 567
Membranous inflammation of the cheek .................... 552
Meninges of brain, fungous tumours 494
Mental disorders, Dr. Uwins on.... 93
Mental derangement affected by the moon .................... 485
Mercury and syphilis, compound of, 166
Mercury, in traumatic inflammation of brain .................. 234
Mercury in the Gibraltar fever .... 348
Mercury in pericarditis .......... 431
Mercury, supposed poisoning by .. 472
Metropolitan Society of General Practitioners................ 270
Metropolitan Society, address of the, 532
Middlemore, Mr. on the eye ...... 459
Mind, Dr. Kennedy on .......... 146
Modern works, mode of propagating 314
Mollescence of the brain.......... 500
Monomaniacal illusions, samples of, 299
Morbid anatomy of diabetes ...... 71
Morphia, Dr. Bardsley on ........ 333
Mortification from burn .......... 501
Moxas for diseases of the heart.... 536
Mucous membrane of bronchi.... 25
Mumps, affecting the testicle...... 399
Murray, Mr. on hydrophobia...... 433

N.
Nævus, subcutaneous, seton for.... 506
National cemetery ............... 480
Neilgherry, climate of the ........ 308
Nerves, Mr. Lizars on division of .. 271
Nervous temperament............. 17
Nervous diseases, treatment of ... 91
Nervous irritation, effects of ...... 328
Neuralgia, from uterine irritation.. 203
Neuralgia of the diaphragm ...... 461
Nux vomica, for amaurosis........ 441

O.
Obliteration of inferior cava ...... 550
O'Brien, Dr. his report on fever ... 408
Obstetric Society, proceedings of the 241
Œsophagus, Mr. Macilwain on .... 200
Œsophagus and trachea, section of 246
Omentum, disease of the.......... 225
Operations on wounded arteries ... 137
Operations for internal strangulations ........................ 544
Opium, immense doses of in hysteria 564
Orfila on poisoning by mercury.... 472
Ossific testis ...................... 406
Os uteri, imperforate, operation for, 553
Ovarian tumours, etiology of...... 47

P.
Pain in the side and spine in hysteria ........................ 36
Pains, sympathetic, in uterine irritation ........................ 41
Pancreas, disease of the .......... 227
Paracentesis thoracis, for empyema, 449
Parisian treatment of intermittents, 154
Pathological anatomy, remarks on, 367
Pathology of the stomach ........ 223
Pearson, Dr. Burton, on brain fever, 198
Penis, sloughing of ............... 436
Perforation of the intestine in fever, 224
Pericarditis, report on............. 183
Pericarditis, from pneumo-thorax .. 189
Pericarditis, anatomical characters of 427
Pericarditis, M. Louis on.......... 429
Pericarditis, mercury in .......... 431
Pericardium, diseases of the ...... 426
Peritonitis, fatal, from cold drink.. 223
Peritonitis, simulated by irritation.. 331
Philalethes on cold lotions ...... 153
Philip, Dr. W. on functional disorders ........................ 311
Phosphate of iron, in diabetes .... 74
Phthisical persons, West Indies for, 81
Phthisis, extraordinary case of .... 529
Phthisis laryngea, case of ........ 543
Physicians, lives of British........ 488
Physiological studies ............ 498
Plethora, observations on ....... 315
Pneumonia, treated by antimony .. 567
Pneumo-thorax, case of .......... 453
Pneumo-thorax, history of........ 455
Poets, are they mad? ............ 300
Poison of hydrophobia............ 435
Poisoning, supposed, by mercury .. 472
Poisoning by opium and belladonna 563
Popliteal aneurism, cases of ...... 283
Popliteal aneurism .............. 365
Posterior tibial wounded ......... 138
Practice, mode of getting into .... 479
Prize essay on temperament...... 1
Proceedings of the Obstetric Society 241
Profession, tax on those entering .. 440
Prolapsus of the uvula............ 540
Puerperal fever, pathology of...... 286
Puerperal fever, suppuration of the veins in ........................ 288
Puffy tumour of scalp from effusion 558
Pulse *not* synchronous with the impulse of the heart............. 128
Purulent depôts in puerperal fever, 287

Q.
Quickening, what is it? .......... 109
Quinine in acute rheumatism...... 437

R.
Rachitome, double .............. 570
Reason, dawn of, in the child ..... 291
Recamier, M. on cold affusion .... 462
Rectum, extirpation of the lower part of........................ 148
Rectum, Mr. Macilwain on........ 193
Rectum, treatment of stricture of.. 194
Reid, Dr. on hypochondriasis...... 83
Reporting in Glasgow ............ 268
Respiratory organs, Mr. Alcock on, 25
Retention of urine .............. 160
Rheumatic pericarditis, fatal cases of 183
Rheumatic metastasis to the bladder 226
Rheumatic pericarditis ........... 428
Rheumatism, acute, quinine in.... 437
Rib, carious, excision of a ........ 554
Ridicule in hypochondriasis ...... 85
Rupture of an aortic aneurism .... 178
Rupture of the spleen, case of...... 247
Ruptured urethra................ 562

S.
St. John Long, inquest on ........ 520
St. John Long, remarks on........ 572
Saline impregnation of the blood .. 219
Salubrity of the Neilgherries ...... 309
Sanity and insanity, affinities of.... 298
Scalp, deceptive depression in the 558
Scarifications, value of........... 546
Scarlatina, followed by empyema.. 447
Scarlet fever, Mr. Alcock on ...... 29
Scarpa's opinions on aneurism .... 358
Scientific pursuits, moderation in .. 88
Scirrhus of rectum, M. Lisfranc on 148
Scirrhus of the lung, case of ...... 520

Scouler, Dr., on production of worms 221
Scrofulous inflammation of testis .. 403
Searle, Mr. on cholera............ 375
Secondary hæmorrhage, amputation 277
Section of trachea and œsophagus.. 246
Serous effusion in old persons .... 171
Seton for subcutaneous nævus .... 506
Sigmoid flexure of colon, stricture of 150
Sigmoid flexure of colon, stricture of 228
Sir Astley Cooper on the testis .... 385
Sloughing of an aneurismal sac.... 362
Sloughing of penis ............... 436
Sloughy abscess of the leg, report on 281
Small-pox, Mr. Alcock on ........ 27
Smith, Dr., his letter to the editor 230
Society, Obstetric, proceedings of.. 241
Spine, pain of the, in hysteria .... 35
Spine, new mode of opening the .. 570
Spleen, fatal hæmorrhage from the 227
Spleen, apoplexy of the .......... 247
Spleen, Sir Anthony Carlisle on the 486
Spleen, a warming pan for the stomach ......................... 487
Spleen, Mr. Dobson on the........ 531
Spleen, a reservoir for the stomach 531
Spleen, experiments on the ...... 531
Steevens, Dr., on the blood........ 217
Stethoscope, new ................ 563
Stimuli in concussion ............ 561
Stomach and intestines, pathology of 223
Stomach, on organic disease of the 319
Stomach, hypertrophy of the ...... 491
Strangulations, operation for...... 544
Strangulations, internal .......... 568
Stricture of the rectum .......... 193
Stricture of the œsophagus ........ 200
Strychnine, amaurosis treated by .. 441
Strychnine in amaurosis .......... 460
Subclavian tied for hæmorrhage.... 500
Submaxillary nerves, division of the 271
Suppression of habitual evacuations 467
Swan Alley sore.................. 167
Syme, Mr. on the excision of joints 273
Syncope, an effect of loss of blood.. 60
Syncope from hæmorrhage........ 463
Syphilis, Mr. Travers on.......... 165
Syphilis and mercury, compound of 166

T.

Tate, Mr., on hysteria............ 30
Tax on those entering the profession 440
Temperament, prize essay on...... 1
Testis, Sir Astley Cooper on ...... 385
Testis, diseased, case of .......... 457
Testis, M. Larrey on diseases of the 540
Tetanus, Dr. Adams on .......... 476
Thomas, Dr. his physiologie des temperamens .................... 1
Thompson, Dr., on clouds ........ 475
Thorax, paracentesis of .......... 449
Thorax, fungous tumour of the .... 517
Throat, hæmorrhage from ........ 260
Tibial nerve, inflammation of the.. 281
Tonnelé, M., on puerperal fever .. 286
Tonsils, calomel in inflammation of 484
Trachea and œsophagus, section of 246
Tracheotomy...................... 464
Travers, Mr., on venereal affections 162
Trismus traumaticus, case of...... 458
Typhoid fever, types of .......... 409
Typhus fever, blood-letting in...... 414
Typhus fever, Dr. O'Brien on...... 414

U.

Ulceration of the throat, fatal .... 260
Ulnar artery wounded—operation.. 139
Union by the first intention ...... 190
University of London, diploma of .. 204
Urethra, ruptured, new treatment of 562
Urinary flux of children, cases of .. 75
Uterine irritation, Dr. Addison on.. 40
Uterine irritation, cold injections in 41
Uterine irritation, case of ........ 203
Uvula, prolapsus of the .......... 540
Uwins, Dr., on mental disorders .. 93

V.

Vaginal discharges .............. 417
Vaginal discharges in children .... 422
Vagina, imperforate, fatal operation 466
Vapour bath for sloughing of penis 436
Varicose aneurism, M. Dupuytren on 509
Venables, Dr., on diabetes........ 68
Venereal affections, Mr. Travers on 162
Venereal testis .................. 405
Veratria, Dr. Bardsley on ........ 336
Vomica opening externally........ 529

W.

Wakley on diseases of the heart! .. 471
Wasting of the testis ............ 391
West Indies, climate of the........ 79
Worms in the large bowels........ 213
Worms, production of, in the body 221
Wounded arteries, Mr. Guthrie on.. 133
Wright, Dr., his medical report.... 173

Y.

Yellow fever, cause of, in the blood 217
Youatt, Mr., on hydrophobia...... 433

### CORRESPONDENCE.

A Physician residing in the vicinity of one of the hospitals wishes to receive into his family two House Pupils, who will have ample opportunities of becoming practically acquainted with their profession.

The Physician is well known to the Editor of this Journal, to whom applications (if by letter post paid) may be made. Terms—120 Guineas a Year.

